# A challenging TSH/GH co-secreting pituitary adenoma with concomitant thyroid cancer; a case report and literature review

**DOI:** 10.1186/s12902-021-00839-x

**Published:** 2021-08-30

**Authors:** Jee Hee Yoon, Wonsuk Choi, Ji Yong Park, A Ram Hong, Sung Sun Kim, Hee Kyung Kim, Ho-Cheol Kang

**Affiliations:** 1 Department of Internal Medicine, Chonnam National University Medical School, 160, Baekseo-ro, Dong-gu, 61469 Gwangju, South Korea; 2 Department of Pathology, Chonnam National University Medical School, 160, Baekseo-ro, Dong-gu, 61469 Gwangju, South Korea

**Keywords:** Thyroid stimulating hormone (TSH) secreting pituitary adenoma (TSHoma), Acromegaly, Thyroid cancer, Coexistence, Complications

## Abstract

**Background:**

Thyroid stimulating hormone (TSH) secreting pituitary adenoma (TSHoma) with coexisting thyroid cancer is extremely rare, and proper treatment of both diseases may pose a unique clinical challenge. When TSHoma has plurihormonality, particularly involving the co-secretion of growth hormone (GH), management can be more complicated. Herein, we present a difficult-to-manage case of papillary thyroid cancer with an incurable TSH/GH-secreting pituitary adenoma.

**Case presentation:**

A 59-year-old man was referred to our hospital due to memory impairment and inappropriate TSH level. Sella magnetic resonance imaging revealed a huge pituitary mass extending to the suprasellar area. Clinical diagnosis of TSH/GH co-secreting pituitary adenoma was made based on elevated free T4, total T3, serum α-subunit, insulin-like growth factor-1 levels and non-suppressible GH levels after oral glucose loading. Rectal cancer and multifocal papillary thyroid microcarcinoma (PTMC) were diagnosed during initial screening for internal malignancy; lower anterior resection was performed and close observation was planned for PTMC. Long-acting octreotide therapy was commenced, which resulted in a dramatic reduction in TSHoma size and facilitated control of hormonal excess. Total thyroidectomy and radioactive iodine (RAI) therapy were needed during follow up due to the growth of PTMC. After the surgery, the pituitary adenoma represented resistance to somatostatin analogue therapy and the tumor size gradually increased despite the addition of dopamine agonist therapy. Furthermore, TSH suppressive therapy with levothyroxine was impossible and an adequate TSH level for RAI therapy was unmountable. Late debulking pituitary surgery was ineffective, and the patient gradually deteriorated and lost to follow up.

**Conclusion:**

We report the first aggravated case of TSH/GH co-secreting pituitary tumor after total thyroidectomy for concomitant multifocal PTMC. Deferring of thyroid surgery until the TSHoma is well controlled may be the optimal therapeutic strategy in patients with TSHoma and coexistent thyroid cancer; ablative thyroid surgery may result in catastrophic pituitary tumor growth.

**Supplementary Information:**

The online version contains supplementary material available at 10.1186/s12902-021-00839-x.

## Background

Thyroid stimulating hormone (TSH)-secreting pituitary adenomas (TSHomas) are less than 1% of pituitary adenomas, however it has been reported more frequently in recent decades due to increased screening tests and clinical awareness [1]. Up to 30% of TSHomas represent plurihormonality and the most common co-secreting hormone is growth hormone (GH) [2]. The diagnostic approach and management of TSHoma with plurihormonality are challenging. Firstly, clinical features of hormonal excess could overlap or be hidden, which makes misdiagnosis or delayed diagnosis. Additionally, plurihormonal pituitary adenomas reveal greater tumor recurrence and local invasion than do mono-secreting pituitary adenomas [3]. Lastly, increased rates of various cancers and cardiovascular complications are predicted, which is exaggerated if co-secreting pituitary hormone is GH [4–6]. Plurihormonal TSHoma with concomitant thyroid cancer developed by chronic hypersecretion of TSH is extremely rare, and its management is more complicated [7, 8]. Herein, we report our experience in treating a case of papillary thyroid cancer (PTC) with incurable TSH/GH-secreting pituitary adenoma and discuss the therapeutic strategy and follow-up.

## Case presentation

A 59-year-old man was referred to our hospital for further evaluation of an inappropriate TSH level that had been detected at local clinic during a workup for dementia due to recurrent memory loss at local clinic. He complained of chronic fatigue, recently decreased visual acuity, and loss of libido. The thyroid function test revealed an increase in serum TSH level to 5.41 μIU/mL (reference range: 0.4–4.8), despite elevated level of free thyroxine (FT4) 3.21 ng/dL (reference range: 0.8–1.71 ng/dL) and total triiodothyronine (T3) 289 ng/dL (reference range: 6o–160 ng/dL) levels. Thyroid autoantibody tests, including of the anti-thyroid peroxidase antibody, anti-thyroglobulin antibody, and TSH-binding inhibitory immunoglobulin, were negative. He had no signs or symptoms of thyrotoxicosis, and a physical examination revealed no goiter. He had no significant medical history, including thyroid disease or head and neck irradiation. His elder brother had thyroid cancer, but thyroid function was normal before the surgery.

**Fig. 1:**
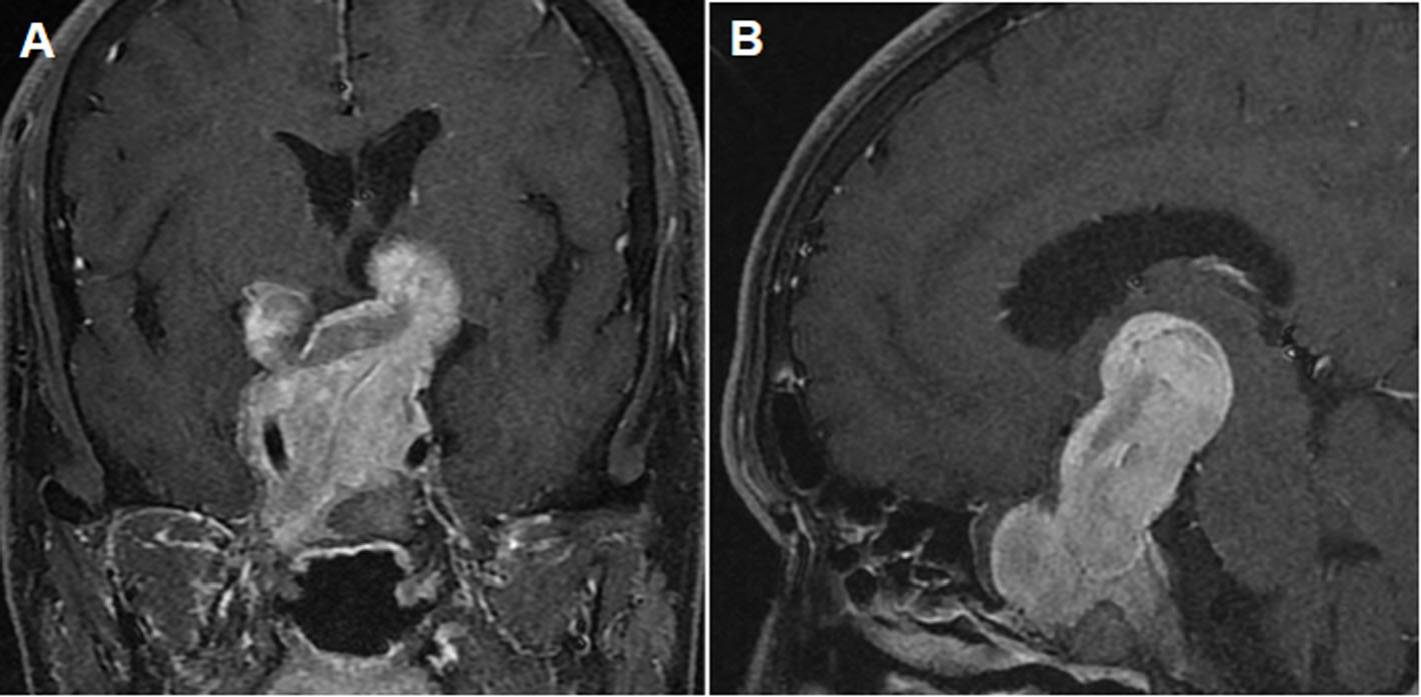
Sella MRI revealed a 7.0 × 6.2 × 5.1 cm sized lobulated heterogeneously enhancing pituitary tumor with supra-sellar expansion. **A** Coronal post-contrast T1 weight image; **B** Sagittal post-contrast T1 weight image

Additional hormonal and imaging studies were performed to distinguish between TSH-secreting pituitary adenoma and thyroid hormone resistance syndrome. The serum α-subunit level was 1.88 mIU/mL (reference range: 0–0.8 mIU/mL) and sellar magnetic resonance imaging (MRI) revealed a 7.0 cm, multi-lobulated heterogeneously enhanced pituitary mass with invasion of the cavernous sinuses and supra-sellar extension (Fig. 1). Thyroid hormone receptor-beta gene mutation was not detected. A pituitary basal hormone study showed an elevated level of insulin-like growth factor-1 (IGF-1) 478.61 ng/mL (age-matched reference range: 71–263 ng/mL); the other pituitary hormones were within normal limits (Supplementary Table 1). A GH suppression test with 75 g oral glucose revealed no suppression of GH (GH nadir, 1.3 ng/mL). He was diagnosed with a TSH/GH-secreting pituitary adenoma. An ophthalmological examination revealed bitemporal hemianopsia (Supplementary Fig. 1). Surgical resection was considered the best treatment option; however, curative pituitary surgery, which the patient refused, was impossible due to invasion. We decided to start long-acting octreotide to control the abnormal TSH and GH secretions.

Positron emission tomography-computed tomography (PET-CT) was performed to screen for malignancy associated with the overproduction of TSH and GH; it showed benign hepatic cysts up to 3.5 cm and ^18^F-fluorodexoyglucose (FDG) avid lesions on the thyroid gland and sigmoid colon. Thyroid ultrasonography (US) revealed a 0.5 cm spiculated hypoechoic nodule with microcalcification, and fine needle aspiration revealed PTC. We decided to closely observe the thyroid cancer without surgery due to the small sized tumor (< 1 cm), no suspicious lymph nodes on US and staging neck CT, and our concerns for potential rapid growth of the pituitary tumor after surgical removal of the thyroid gland. Follow-up thyroid US at 11 months revealed an increase in tumor size from 0.3 × 0.5 × 0.4 cm to 0.7 × 0.5 × 0.5 cm so, surgery was considered. The patient underwent total thyroidectomy with central lymph node dissection; BRAF V600E-positive multifocal PTCs and metastatic cervical lymph nodes (LNs; 7 of the 11 resected LNs) were confirmed pathologically. A metastatic paratracheal LN measuring 8 mm in the largest diameter had extra-nodal extension. The final TNM stage was pT1aN1aM0 (stage II). After surgery, it was impossible to suppress the TSH sufficiently, despite 400 μg levothyroxine, to achieve a goal of the middle to upper half of the reference range for free T4 in a short period immediately after surgery. Furthermore, the TSH level increased insufficiently after levothyroxine withdrawal in preparation for RAI therapy (TSH, 12.22 μIU/mL). On RAI therapy with dose of 180 mCi, off-Tg level was less than 1 ng/ml, and post-treatment scan showed strong RAI uptake in anterior neck, both thyroid beds, and right upper mediastinum. After RAI therapy, levothyroxine (200–250 μg/day) replacement was continued and there was no PTC recurrence during the next 47 months.

**Fig. 2:**
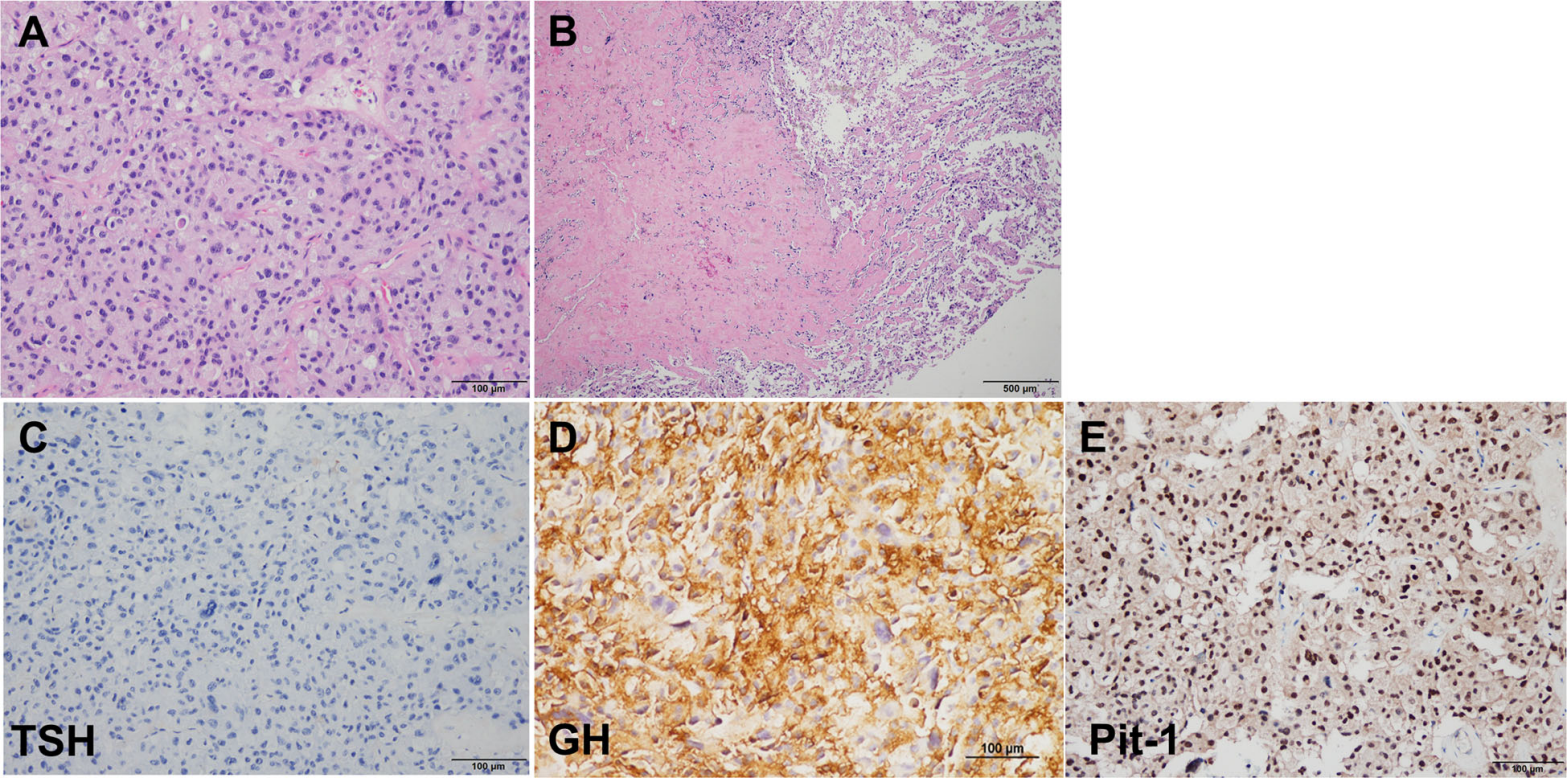
Pathologic finding of pituitary tumor in a patient with TSH/GH co-secreting tumor: Microscopic features reveal large polygonal shaped tumor cells with occasional pleomorphic nuclei. They are arranged in a trabecular or diffuse pattern. **A** Hematoxylin and eosin stain(× 200). **B** Hematoxylin and eosin stain(× 40), Fibrotic nodular area. **C** Immunohistochemical stain for TSH (× 200). **D** Immunohistochemical stain for GH (× 200). **E** Immunohistochemical stain for Pit-1 (× 200)

**Fig. 3:**
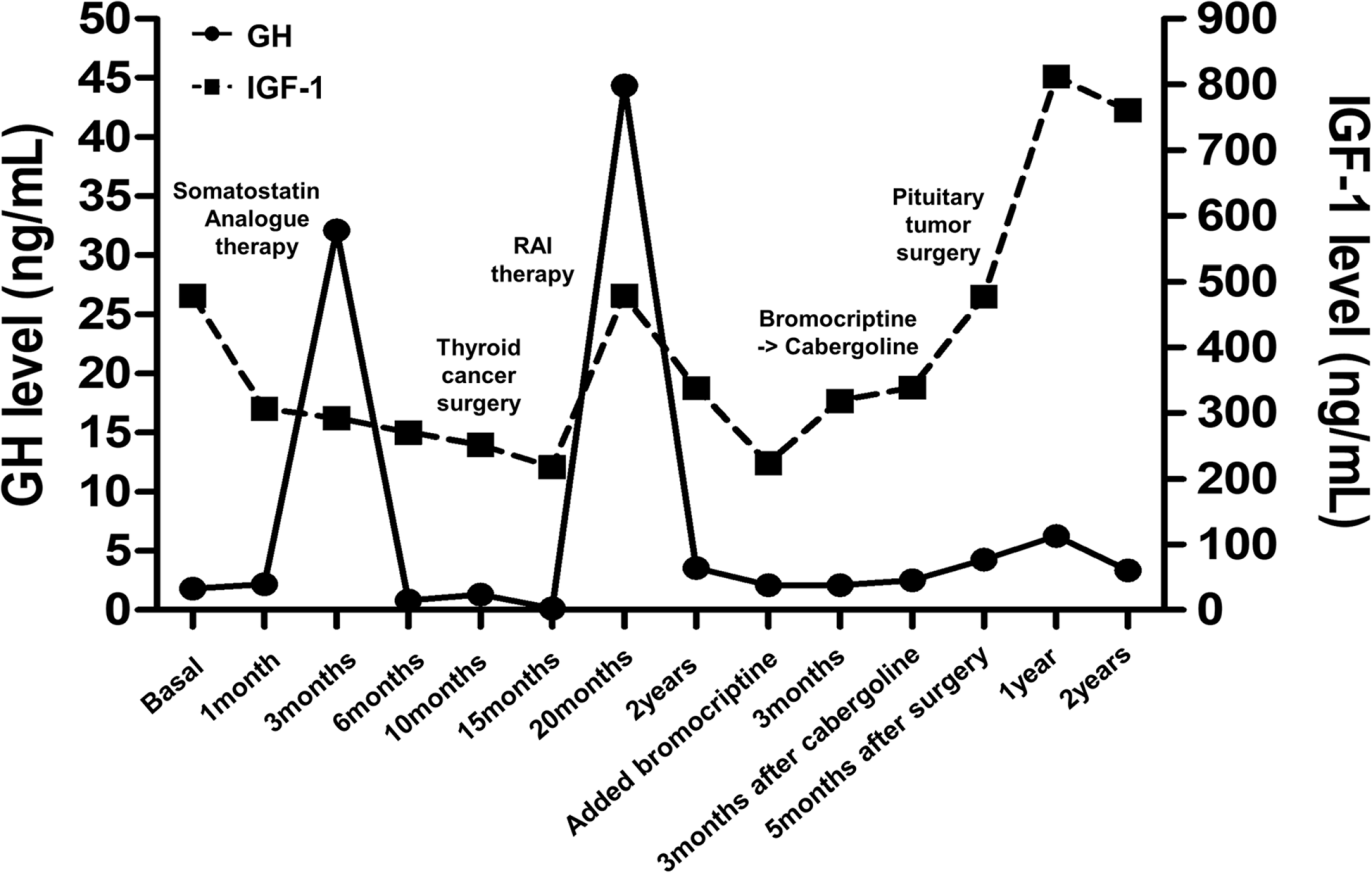
The change of serum GH and IGF-1 according to treatment of TSHoma and coexist thyroid cancer. Reference range (age and sex matched): IGF-1, 71–290 (ng/mL). IGF-1: insulin-like growth factor-1

**Fig. 4:**
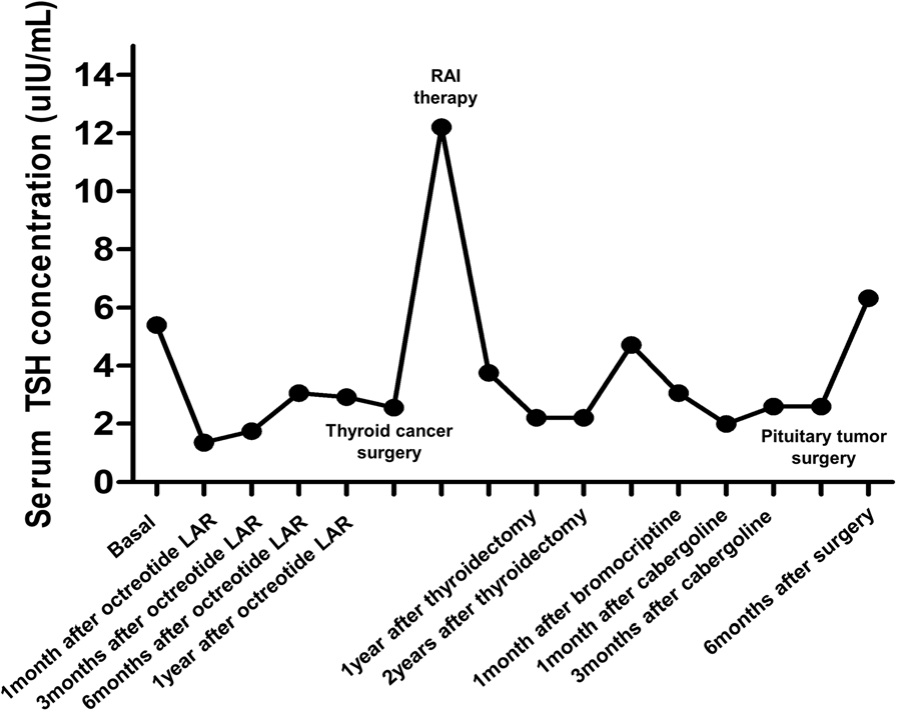
The change of serum TSH concentration according to treatment of TSHoma and coexist thyroid cancer. Reference range: TSH, 0.4–4.8 (uIU/mL). TSH: thyrotropin; LAR: long acting release

Sellar MRI, performed at the 6-month follow-up after octreotide LAR therapy (before thyroid surgery), revealed a reduction in the size of the pituitary adenoma to 5.6 cm. Additionally, GH and IGF-1 levels were controlled to 0.15 ng/mL and 217.74 ng/mL, respectively. However, the size of the tumor increased to 6.4 cm, and serum GH and IGF-1 levels increased to 44.34 ng/mL and 507.77 ng/mL, respectively, 6 months after RAI therapy. Despite continued long-acting octreotide therapy, his symptoms worsened. Decompressive pituitary surgery was attempted, but the tumor removal was impossible due to the hard and fibrotic nature of the tumor and excessive bleeding. Pathologic specimen confirmed the diagnosis of Pit-1 positive TSH/GH secreting pituitary adenoma (Fig. 2). The somatostatin analogue therapy was maintained for 2 years after surgery and cabergoline was added on, but GH and IGF-1 levels remained in high range with continued tumor growth. The patient was lost to follow up 3 months after discontinuance of somatostatin analogue therapy. The changes of serum TSH and GH levels during treatment of the patient are described in Figs. 3 and 4.

## Discussion and conclusion

TSHomas comprise the smallest portion of pituitary adenomas, and concurrent thyroid cancer is extremely rare; only a few case reports and small series have been published [9]. Thyroid hormone secretion from TSHomas is autonomous [10, 11]. Patients with TSHoma present with an inappropriate serum TSH level, with elevated serum free T3 and FT4 [5]. The thyroid gland in TSHoma patients is stimulated by persistently high and uncontrolled TSH levels, which provide a good environment for the development of thyroid nodules and cancer [12]. Most TSHoma patients develop thyroid goiter and multiple thyroid nodules [13]. Perticone et al. [14] reported that 3 of 62 patients (4.8%) with TSHoma also had thyroid cancer. Thus, TSHoma coexisting with thyroid cancer has a non-negligible incidence and is a complex disease that is challenging to treat. Furthermore, when a TSHoma co-secretes other pituitary hormones, its management becomes even more difficult because of the interactions among hormones [15]. Approximately one-quarter of TSHomas are mixed pituitary adenomas with concomitant hormonal hypersecretion; TSH/GH co-secretion is the most frequent [5, 16]. Only two cases of TSH/GH-secreting pituitary adenoma with simultaneous thyroid cancer have been reported; both cases were successfully managed by long-acting octreotide therapy and thyroidectomy [17, 18]. However, our case of a TSH/GH-secreting pituitary adenoma and concomitant thyroid cancer was difficult to treat.

Early diagnosis is important for proper management of TSHoma with plurihormonality, but hormonal interactions can cause delayed diagnosis by masking clinical clues. TSHoma leads to secondary hyperthyroidism, so thyroid goiter or hyperthyroidism are also observed in most TSHoma patients [8, 19–21]. However, our patient did not show any symptoms or signs of a thyrotoxicosis. Also, he did not exhibit overt physical manifestations of acromegaly, although an elevated IGF-1 level and lack of GH suppression during the oral glucose challenge test represented GH co-secretion. In previous studies, the clinical signs and symptoms of thyrotoxicosis or GH excess can be missed in patients with TSH/GH co-secreting pituitary adenoma [5, 22]. Simultaneous overproduction of TSH and GH can mask the hypersecretion of these hormones. The delayed diagnosis in our case, due to unclear clinical manifestations of TSH and GH overproduction by the pituitary tumor, led to us encountering a huge incurable TSH/GH-secreting tumor; this tumor was difficult to treat due to concurrent thyroid cancer. Hypersecretion of TSH and GH may provide conditions that are conducive to tumor proliferation [23–25]. Our patient had thyroid cancer and rectal cancer, which are related to TSH and GH hypersecretion, respectively.

Surgical resection is the treatment of choice in most cases of TSHoma and acromegaly to reduce the risks of co-morbidities and mortality caused by excess TSH and GH [5, 26]. Successful medical management with either a somatostatin analogue or a dopamine agonist has been reported in patients with inoperable tumors and in patients who decline surgery [17, 27]. When a TSHoma co-occurs with thyroid cancer, optimal management is challenging. The clinical courses of TSHoma with coexisting thyroid cancer including our cases are summarized in Table 1. The order of priority with respect to the treatment of the pituitary tumor and thyroid cancer remains controversial. In the past, earlier management of thyroid cancer was preferred because it is a malignancy [17, 28]; however, a high incidence of invasiveness of large TSHomas has been reported among patients with a previous history of thyroidectomy or RAI therapy [5]. Prior studies have suggested that suppression of the normal negative feedback mechanism via removal of the thyroid gland could promote pituitary tumor growth in patients with TSHoma [23, 29]. In our case, the pituitary tumor decreased in size and the IGF-1 level also decreased up to 15 months after starting long-acting octreotide; however, the pituitary tumor regrew, and elevated serum IGF-1 levels were observed after thyroidectomy and RAI therapy. Although the degree of TSHoma regression could not be measured by a thyroid function test, elevated GH and IGF-1 levels were demonstrated in relation to activity in the hypothalamic-pituitary-thyroid axis; however, the mechanism remains unclear [30, 31]. Therefore, curative surgery or aggressive management of TSH/GH-secreting pituitary adenoma is needed before thyroidectomy. The changes in the visual field test result correlated with tumor regression and regrowth, which could be helpful to estimate the improvement of pituitary tumor treatment.

**Table 1:** Clinical characteristics of TSHomas with concurrent thyroid cancer reported in literature and present cases

Reference	Age/Sex	Pituitary tumor		Thyroid cancer			Pre-op management	The order of surgeries		Radio-iodine ablation therapy Preparation (dose)	TSH level at the last follow up	Clinical outcome	
		Size (cm)	Secreting hormone	Histologic type	Size (cm), multifocality	TNM stage		1st surgery	2nd surgery			Pituitary tumor	Thyroid cancer
Calle-Pascual [7] et al	55/M	NR	TSH	FTC	5.0	NR	No	TT	TSA&TR	NR (35 mCi)	Undetectable on LT4	Remission (1 year)	Remission (1 year)
Gasparoni [19] et al	37/F	1.0	TSH	PTC,	2.0	T2N0M0	No	TT	No surgery for pituitary tumor	NR (150 mCi)	NR	Remission (NR)	NR
Kishida [21] et al	27/F	1.0	TSH	PTC	3.0	T2N1M0	Octreotide	TSA&TR	Lobectomy	No	Normal range without LT4	NR	Remission (9 months)
Ohta [18] et al	45/F	1.5	TSH	PTC	2.0	NR	Octreotide	TT	TR via right pterional approach	No	Normal range	Remission (4 months)	NR
Gessl [20] et al	22/F	0.4	TSH/PRL	FTC	0.8	T1N0M0	No	TT	TSA&TR	NR (80 mCi)	0.6 mU/L	Remission (NR)	NR
Poggi [8] et al	50/M	0.3	TSH	FTC	1.7	NR	No	TT	No surgery for pituitary tumor	No	6.97 uIU/mL	NR	Remission (12 months)
Nguyen [17] et al	57/F	2.6	TSH/GH	PTC	0.8, multifocal	NR	Octreotide	TT	No surgery for pituitary tumor	rhTSH (100 mCi)	0.145 mIU/L	Remission (20 months)	Octreotide response
Ünlütürk [23] et al	38/F	2.2	TSH	PTC, Oncocytic variant	4.0, multifocal	T3N1M0	Octreotide	TT	TSA&TR - > gamma knife surgery	NR (150 mCi)	3.4 ~ 3.7 mU/L	Stable residual tumor	Remission (84 months)
	27/F	2.8	TSH	PTC	1.0, multifocal	T1N0M0	Methimazole	TT	None	NR (150 mCi)	4.7 mU/L	Residual tumor on lanreotide	Remission (6 months)
Perticone [14] et al	47/M	1.9	TSH	FTC, Hürthle cell variant	1.5	T1bN0M0	Lanreotide	TT	TSA&TR	rh TSH (106 mCi)	0.1 mU/L	Remission (6 months)	Remission (1 year)
	46/M	0.7	TSH	PTC, Follicular variant	1.2	T1bN0M0	Lanreotide	TSA&TR	TT	rh TSH (100 mCi)	0.2	Remission (5 years)	Remission (5 years)
	42/M	1.2	TSH	PTC	NR, multifocal	T3bN1aM0	No	TT	TSA&TR	NR (100 mCi)	< 0.1 and 0.3	Remission (28 months)	Remission (28 months)
Present case	59/M	7.0	TSH/GH	PTC	0.5, multifocal	T1aN1aM0	Octreotide	TT	TR via interhemispheric approach	Levothyroxine withdrawal (180 mCi)	1.99 uIU/ml	Residual tumor on lanreotide	Remission (44 months)

*TSH* thyroid stimulating hormone, *GH* growth hormone, *PRL* prolactin, *PTC* papillary thyroid cancer, *FTC* follicular thyroid cancer, *TT* total thyroidectomy, *TSA & TR* trans-sphenoidal approach and tumor removal, *NR* not reported, *rh* recombinant human

In our patient, surgical specimen of pituitary tumor represented fibrotic change, marked nuclear pleomorphism, and negative TSH staining. Long-term treatment with octreotide LAR might be related to the fibrotic change and TSH-negativity in pituitary tumor. Yamada et al [32] reported that negative TSH staining was observed in 3 of 90 patients who underwent pituitary surgery for TSHoma. In our case, TSH staining was still not observed; even though additional staining was performed and a positive Pit-1 staining was demonstrated. Scarcely reported Pit-1 lineage plurihormonal pituitary adenomas are frequently large and invasive. Pit-1 overexpression was associated with cell proliferation in GH, prolactin, and TSH secreting pituitary adenomas, even though the pathomechanism was still unclear [15, 33].

The conventional treatments for differentiated thyroid cancer (DTC) are surgery, radioactive iodine (RAI) therapy, and TSH suppression therapy [34–36]. Other challenges after the thyroid surgery were encountered. Autonomous TSH secretion prevents achievement of adequate TSH levels for RAI and thyroxine suppression therapy. Stimulation with recombinant TSH (rhTSH) and conventional thyroid hormone withdrawal are used to elevate the serum TSH level [35]. Several studies used rhTSH, rather than levothyroxine withdrawal, in TSHoma patients for RAI therapy to avoid pituitary tumor growth and achieve a sufficient TSH level for promoting efficacious therapy [14, 17, 23]. In our patient, levothyroxine withdrawal was used to prepare the patient for RAI therapy due to financial constraints; however, his peak TSH level (12.22 mIU/mL) was not sufficient for optimal therapy. At the time of RAI therapy, multifocal PTCs and multiple lymph node metastases were indications for therapeutic RAI therapy and high dose RAI therapy was administered because of concerns regarding an unreliable TSH level and difficulty of follow up [35]. After RAI therapy, the serum TSH level of our patient was not suppressed below 1.99 mIU/mL, even though thyroid hormone replacement was sufficient. The use of rhTSH to prepare a patient for RAI therapy is optimal in terms of achieving an appropriate TSH level while avoiding the possibility of TSHoma growth (unlike levothyroxine withdrawal). Furthermore, the serum TSH level does not reflect the thyroid status or the severity of TSHoma in cases where the TSHoma is not treated curatively. Further studies are necessary to identify a better method to evaluate thyroid functional status and the extent of TSH secretions by TSHomas.

In conclusion, our case involved a large Pit-1 lineage TSH/GH co-secreting pituitary macroadenoma with concomitant thyroid cancer was challenging to diagnose and treat. Controlling the pituitary tumor before thyroidectomy is important to avoid rapid rebound of pituitary tumor growth due to the attenuation of thyroid gland-mediated negative feedback. An appropriate thyroid cancer management strategy, such as using rhTSH to prepare the patient for RAI therapy followed by meticulous surveillance after thyroid surgery, is necessary for the successful treatment of TSHoma and concomitant thyroid cancer.

## Supplementary Information

**Additional file 1: Supplementary table 1.** Baseline pituitary hormone test

**Additional file 2: Supplementary figure 1.** The change of the visual field examination. A: At the time of the first diagnosis of TSH/GH co-secreting tumor. B: Pre-operative work up before thyroid cancer surgery

## Data Availability

All the data generated and/or analyzed during this study are included in this article.

## Abbreviations

FDG: Fluorodeoxyglucose
GH: Growth hormone
IGF-1: Insulin-like growth factor-1
MRI: Magnetic resonance imaging
PET-CT: Positron emission tomography-computed tomography
PTMC: Papillary thyroid microcarcinoma
RAI: Radioactive iodine
T3: Triiodothyronine
T4: Thyroxine
TSH: Thyroid stimulating hormone
US: Ultrasonography

## References

[1] Beck-Peccoz P, Persani L, Lania A, De Groot LJ, Beck-Peccoz P, Chrousos G, Dungan K, Grossman A, Hershman JM, Koch C, McLachlan R, New M, Rebar R (2000). Thyrotropin-secreting pituitary adenomas. Endotext.

[2] Beck-Peccoz P, Brucker-Davis F, Persani L, Smallridge RC, Weintraub BD (1996). Thyrotropin-secreting pituitary tumors. Endocr Rev.

[3] Nishioka H, Inoshita N (2018). New WHO classification of pituitary adenomas (4th edition): assessment of pituitary transcription factors and the prognostic histological factors. Brain Tumor Pathol.

[4] Jenkins PJ (2006). Cancers associated with acromegaly. Neuroendocrinology.

[5] Beck-Peccoz P, Persani L, Mannavola D, Campi I (2009). Pituitary tumours: TSH-secreting adenomas. Best Pract Res Clin Endocrinol Metab.

[6] Lombardi G, Di Somma C, Grasso LF, Savanelli MC, Colao A, Pivonello R (2012). The cardiovascular system in growth hormone excess and growth hormone deficiency. J Endocrinol Investig.

[7] Calle-Pascual AL, Yuste E, Martin P, Aramendi T, Garcia-Maurino ML, Argente J, Catalan MJ, Uria J, Cabranes JA, Charro AL (1991). Association of a thyrotropin-secreting pituitary adenoma and a thyroid follicular carcinoma. J Endocrinol Investig.

[8] Poggi M, Monti S, Pascucci C, Toscano V (2009). A rare case of follicular thyroid carcinoma in a patient with thyrotropin-secreting pituitary adenoma. Am J Med Sci.

[9] Kesmodel SB, Terhune KP, Canter RJ, Mandel SJ, LiVolsi VA, Baloch ZW, Fraker DL (2003). The diagnostic dilemma of follicular variant of papillary thyroid carcinoma. Surgery.

[10] Foppiani L, Del Monte P, Ruelle A, Bandelloni R, Quilici P, Bernasconi D (2007). TSH-secreting adenomas: rare pituitary tumors with multifaceted clinical and biological features. J Endocrinol Investig.

[11] Ness-Abramof R, Ishay A, Harel G, Sylvetzky N, Baron E, Greenman Y, Shimon I (2007). TSH-secreting pituitary adenomas: follow-up of 11 cases and review of the literature. Pituitary.

[12] Fiore E, Vitti P (2012). Serum TSH and risk of papillary thyroid cancer in nodular thyroid disease. J Clin Endocrinol Metab.

[13] Beck-Peccoz P, Giavoli C, Lania A (2019). A 2019 update on TSH-secreting pituitary adenomas. J Endocrinol Investig.

[14] Perticone F, Pigliaru F, Mariotti S, Deiana L, Furlani L, Mortini P, Losa M (2015). Is the incidence of differentiated thyroid cancer increased in patients with thyrotropin-secreting adenomas? Report of three cases from a large consecutive series. Thyroid.

[15] Ng HY, Namboodiri D, Learoyd D, Davidson A, Champion B, Preda V (2019). Clinical challenges of a co-secreting TSH/GH pituitary adenoma. Endocrinol diabetes Metab Case Rep.

[16] Mantovani G, Asteria C, Pellegrini C, Bosari S, Alberti L, Bondioni S, Peverelli E, Spada A, Beck-Peccoz P (2006). HESX1 expression in human normal pituitaries and pituitary adenomas. Mol Cell Endocrinol.

[17] Nguyen HD, Galitz MS, Mai VQ, Clyde PW, Glister BC, Shakir MK (2010). Management of coexisting thyrotropin/growth-hormone-secreting pituitary adenoma and papillary thyroid carcinoma: a therapeutic challenge. Thyroid.

[18] Ohta S, Nishizawa S, Oki Y, Namba H (2001). Coexistence of thyrotropin-producing pituitary adenoma with papillary adenocarcinoma of the thyroid--a case report and surgical strategy. Pituitary.

[19] Gasparoni P, Rubello D, Persani L, Beck-Peccoz P (1998). Unusual association between a thyrotropin-secreting pituitary adenoma and a papillary thyroid carcinoma. Thyroid.

[20] Gessl A, Vierhapper H, Feichtinger H (2006). Non-suppressible TSH in a patient thyroidectomized due to follicular thyroid carcinoma. Exp Clin Endocrinol Diabetes.

[21] Kishida M, Otsuka F, Kataoka H, Yokota K, Oishi T, Yamauchi T, Doihara H, Tamiya T, Mimura Y, Ogura T (2000). Hyperthyroidism in a patient with TSH-producing pituitary adenoma coexisting with thyroid papillary adenocarcinoma. Endocr J.

[22] Johnston PC, Hamrahian AH, Prayson RA, Kennedy L, Weil RJ (2015). Thyrotoxicosis with absence of clinical features of acromegaly in a TSH- and GH-secreting, invasive pituitary macroadenoma. Endocrinol Diabetes Metab Case Rep.

[23] Unluturk U, Sriphrapradang C, Erdogan MF, Emral R, Guldiken S, Refetoff S, Gullu S (2013). Management of differentiated thyroid cancer in the presence of resistance to thyroid hormone and TSH-secreting adenomas: a report of four cases and review of the literature. J Clin Endocrinol Metab.

[24] Kim HK, Lee JS, Park MH, Cho JS, Yoon JH, Kim SJ, Kang HC (2014). Tumorigenesis of papillary thyroid cancer is not BRAF-dependent in patients with acromegaly. PLoS One.

[25] Petroff D, Tonjes A, Grussendorf M, Droste M, Dimopoulou C, Stalla G, Jaursch-Hancke C, Mai M, Schopohl J, Schofl C (2015). The incidence of Cancer among acromegaly patients: results from the German acromegaly registry. J Clin Endocrinol Metab.

[26] Dekkers OM, Biermasz NR, Pereira AM, Romijn JA, Vandenbroucke JP (2008). Mortality in acromegaly: a Metaanalysis. J Clin Endocrinol Metab.

[27] Atkinson JL, Abboud CF, Lane JI (2005). Dramatic volume reduction of a large GH/TSH secreting pituitary tumor with short term Octreotide therapy. Pituitary.

[28] Safer JD, Colan SD, Fraser LM, Wondisford FE (2001). A pituitary tumor in a patient with thyroid hormone resistance: a diagnostic dilemma. Thyroid.

[29] Beck-Peccoz P, Persani L (2002). Medical management of thyrotropin-secreting pituitary adenomas. Pituitary.

[30] Jones PM, Burrin JM, Ghatei MA, O'Halloran DJ, Legon S, Bloom SR (1990). The influence of thyroid hormone status on the hypothalamo-hypophyseal growth hormone axis. Endocrinology.

[31] Laron Z (2003). Interactions between the thyroid hormones and the hormones of the growth hormone axis. Pediatr Endocrinol.

[32] Yamada S, Fukuhara N, Horiguchi K, Yamaguchi-Okada M, Nishioka H, Takeshita A, Takeuchi Y, Ito J, Inoshita N (2014). Clinicopathological characteristics and therapeutic outcomes in thyrotropin-secreting pituitary adenomas: a single-center study of 90 cases. J Neurosurg.

[33] Lee JC, Pekmezci M, Lavezo JL, Vogel H, Katznelson L, Fraenkel M, Harsh G, Dulai M, Perry A, Tihan T (2017). Utility of Pit-1 Immunostaining in distinguishing pituitary adenomas of primitive differentiation from null cell adenomas. Endocr Pathol.

[34] Schlumberger MJ (1998). Papillary and follicular thyroid carcinoma. N Engl J Med.

[35] Mallick UK (2010). The revised American Thyroid Association management guidelines 2009 for patients with differentiated thyroid cancer: an evidence-based risk-adapted approach. Clin Oncol.

[36] Haugen BR, Alexander EK, Bible KC, Doherty GM, Mandel SJ, Nikiforov YE, Pacini F, Randolph GW, Sawka AM, Schlumberger M (2016). 2015 American Thyroid Association management guidelines for adult patients with thyroid nodules and differentiated thyroid Cancer: the American Thyroid Association guidelines task force on thyroid nodules and differentiated thyroid Cancer. Thyroid.

