# Supplementary material for: A challenging TSH/GH co-secreting pituitary adenoma with concomitant thyroid cancer; a case report and literature review
Source: BMC Endocr Disord. 2021 Aug 30;21:177. doi: 10.1186/s12902-021-00839-x (PMC8404254; doi:10.1186/s12902-021-00839-x)
Supplement: Supplementary file 1 — Additional file 1: Supplementary table 1. Baseline pituitary hormone test [file 12902_2021_839_MOESM1_ESM.docx]

|  | Results | Reference range |
| --- | --- | --- |
| LH (IU/L) | 5.83 | 1.5-9.3 |
| FSH (IU/L) | 5.04 | 1.4-18.1 |
| Testosterone (ng/dL) | 480 | 188-896 |
| Prolactin (ng/mL) | 10.81 | 2.8-29.9 |
| ACTH (pg/mL) | 47.2 | 0-60 |
| Cortisol (µg/dL) | 7.2 | 3-23 |
| GH (ng/mL) | 2.1 | 0-10 |

Supplementary table 1. Baseline pituitary hormone test

LH, Luteinizing hormone; FSH, follicle stimulating hormone; ACTH, adrenocorticotropic hormone
