# Supplementary figures and images for: A challenging TSH/GH co-secreting pituitary adenoma with concomitant thyroid cancer; a case report and literature review
Source: BMC Endocr Disord. 2021 Aug 30;21:177. doi: 10.1186/s12902-021-00839-x (PMC8404254; doi:10.1186/s12902-021-00839-x)

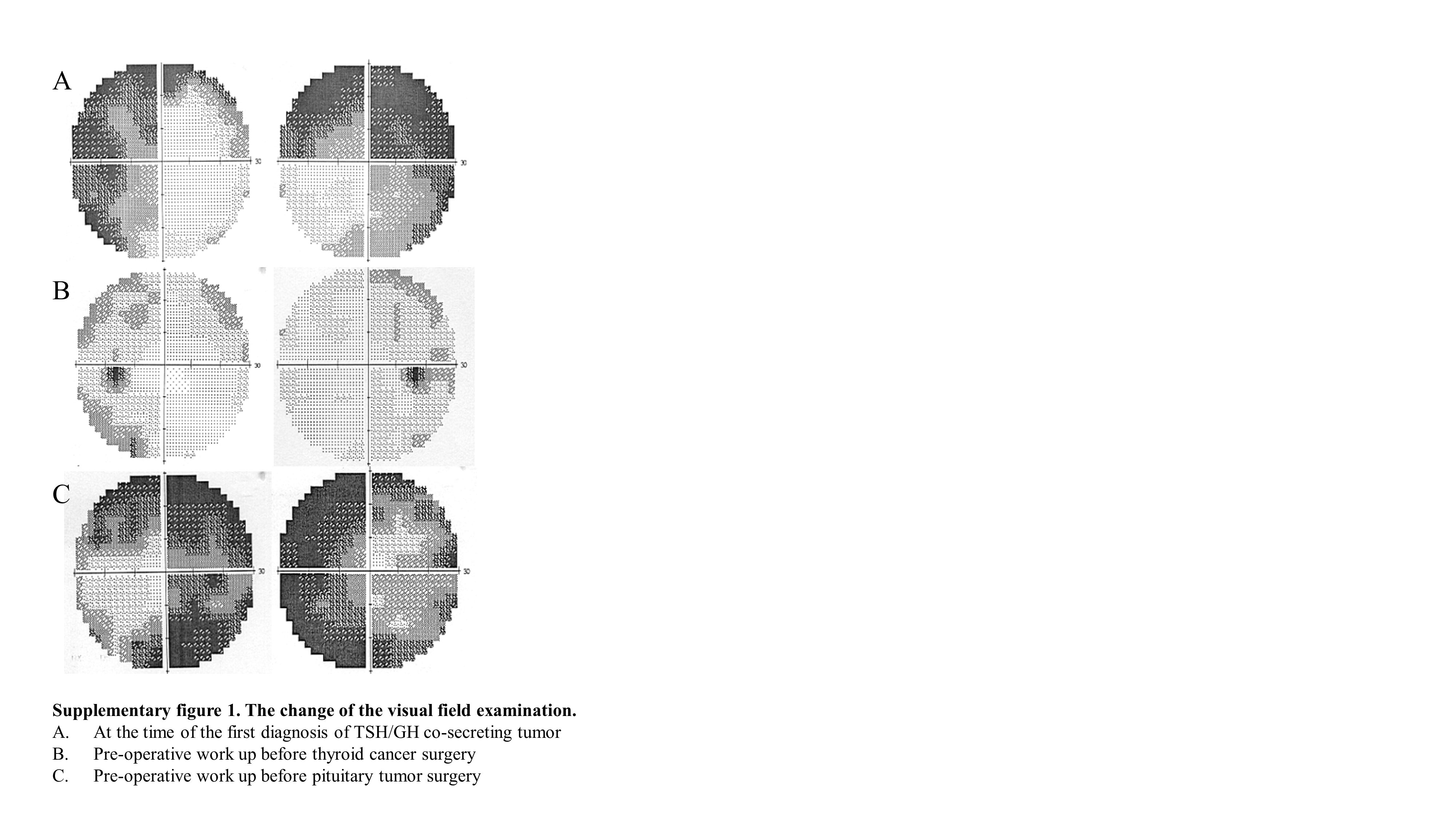

Supplement: Supplementary file 2 — Additional file 2: Supplementary figure 1. The change of the visual field examination. A: At the time of the first diagnosis of TSH/GH co-secreting tumor. B: Pre-operative work up before thyroid cancer surgery [file 12902_2021_839_MOESM2_ESM.tif]
